# Approximation properties of haplotype tagging

**DOI:** 10.1186/1471-2105-7-8

**Published:** 2006-01-09

**Authors:** Staal A Vinterbo, Stephan Dreiseitl, Lucila Ohno-Machado

**Affiliations:** 1 Decision Systems Group, Brigham and Women's Hospital, 75 Francis Street, Boston, MA 02115, USA; 2 Harvard Medical School, Boston, MA, USA; 3 Harvard-MIT, Division of Health Sciences and Technology Boston, MA, USA; 4 Dept. of Software Engineering, Upper Austria University of Applied Sciences, Hagenberg, Austria

## Abstract

**Background:**

Single nucleotide polymorphisms (SNPs) are locations at which the genomic sequences of population members differ. Since these differences are known to follow patterns, disease association studies are facilitated by identifying SNPs that allow the unique identification of such patterns. This process, known as haplotype tagging, is formulated as a combinatorial optimization problem and analyzed in terms of complexity and approximation properties.

**Results:**

It is shown that the tagging problem is NP-hard but approximable within 1 + ln((*n*^2 ^- *n*)/2) for *n *haplotypes but not approximable within (1 - *ε*) ln(*n*/2) for any *ε *> 0 unless NP ⊂ DTIME(*n*^log log *n*^).

A simple, very easily implementable algorithm that exhibits the above upper bound on solution quality is presented. This algorithm has running time *O*((2*m *- *p *+ 1)) ≤ *O*(*m*(*n*^2 ^- *n*)/2) where *p *≤ min(*n*, *m*) for *n *haplotypes of size *m*. As we show that the approximation bound is asymptotically tight, the algorithm presented is optimal with respect to this asymptotic bound.

**Conclusion:**

The haplotype tagging problem is hard, but approachable with a fast, practical, and surprisingly simple algorithm that cannot be significantly improved upon on a single processor machine. Hence, significant improvement in computatational efforts expended can only be expected if the computational effort is distributed and done in parallel.

## Background

Much of the population-wide variation of the human genome can be attributed to single nucleotide polymorphisms (SNPs), which are changes in single base pairs within the genome. SNPs are of specific interest because they allow disease association studies; this means that the involvement of genes in particular diseases can be studied by the analysis of SNP alleles within these genes [1]. For the study of population genomics, SNPs can be used to measure *linkage disequilibrium*, an indication of how much more (or less) likely, compared to chance, certain combinations of neighboring SNP alleles are [2,3].

After the completion of the Human Genome Project emphasized the importance of SNPs to study the location of disease genes, the SNP Consortium project produced a genome-wide map of more than 1.4 million SNPs [4]. Due to linkage disequilibrium, the distribution of possible alleles at SNPs is not uniformly random, and some combinations of neighboring alleles occur more often than others. Such a combination of SNP alleles is called a *haplotype*, and a given set of SNPs can give rise to a wide variety of haplotypes.

It is an important problem to identify a subset of SNPs within a haplotype that allows the unique identification of all possible allele variations within a haplotype. If such a subset can be found, a haplotype can be uniquely identified by knowing only the allele values at a few SNP positions. SNPs that satisfy this requirement are called *haplotype tagging SNPs *(*htSNPs*).

The problem of identifying a minimal set of htSNPs is known to be NP-hard. NP-hardness means that there currently does not exist a way of solving the problem optimally with reasonable computing resources. Even though it is strongly believed among theoretical computer scientists that this state of affairs will prevail, NP-hardness does not exclude the possibility of finding an adequate solution with a reasonable effort. The validity of this last statement hinges on the definitions of the *adequacy *of a solution and what constitutes *reasonable effort*. We will define reasonable effort to be polynomial time computability, while adequacy will be achieved by an approximation algorithm, the solution of which is provably no worse than a certain factor *r *times the optimal.

In recent years, a number of algorithms for calculating htSNPs were developed. Due to the high computational complexity of the problem, these algorithms were either stochastic or, when deterministic, only applicable to haplotypes with a few hundreds to thousands of SNPs. At present, this is not a limitation, since there are few samples that contain these many SNPs. Given the speed of progress and innovation in the biosciences it will, however, be only a matter of time until brute-force approaches will no longer be feasible. Stochastic algorithms do not have this limitation; due to their nature they can give only probabilistic bounds on the results achieved. We believe that approximation algorithms are a viable alternative to both stochastic and brute-force approaches. The advantage over the former is that the bounds on the results are guaranteed to hold; the advantage over the latter is that approximation algorithms can be executed in polynomial time.

The theoretical question of how many SNPs are required to tag a given number of haplotypes was investigated, using Boolean algebra, by Wiuf et al. [5]. The number of htSNPs required for association studies was investigated by Thompson et al. [6]. Previous work on exact algorithms for identifying htSNPs were based on search strategies [7,8]; a stochastic algorithm is given by Johnson et al. [9]. Wiuf et al. [5] and Bafna et al. [10] both present polynomial time algorithms that find a minimum cardinality set of SNPs for the special case that Wiuf et al. call pairwise compatible and Bafna et al. call complete LD, i.e., the four gamete test by Hudson and Kaplan [11] fails for any pair of SNPs. We will in this exposition focus on the general unrestricted case.

## Results

The main results are summarized as follows.

**Theorem 1 ***The haplotype tagging problem is NP-hard and approximable within *1+ln(), *but NP-hard to approximate within c*ln(*n*/2) *for c *> 0 *and not approximable within *(1 - *ε*)ln(*n*/2) *for any ε *> 0 *unless *NP ⊂ DTIME(*n*^log log *n*^). *If we bound n from above by a constant q, the problem is APX-complete*.

We have that *c*ln(*n*/2) = Ω(ln *n*) and 1 + ln((*n*^2 ^- *n*)/2) = *O*(ln *n*), by which we can see that the approximation bound is asymptotically tight. We present an algorithm with running time *O*((2*m *- *p *+ 1)) where *p *= min(*n*, *m*), for the MHT problem that exhibits the above approximation bound. This algorithm is easily implemented, the detailed pseudo code listing presented in this paper contains only 22 lines.

A second algorithm is presented that can be seen as an instance of a schema that lets us approach a family of haplotype tagging problems including the problem of tagging the haplotypes using a set of SNPs with minimal diameter. This latter result addresses the problem of selecting practical "window" size bounds in approaches where such are needed (Bafna et al. [10]).

## Discussion

For 250 million samples (approximately the current population of the USA) the size of the set of tags returned would be at most 39 times larger than the optimum. If we assume that our computer is capable of doing 35 trillion operations per second (the world's currently fastest parallel supercomputer, NEC's Earth Simulator, is capable of this), and we wish to search among 1 million SNPs, the computation using the algorithm presented in this paper would only take about 41 days. If we happen to know that an optimal solution uses at most 1000 of these SNPs (28 is an absolute lower bound for 250 million samples), we would be done in about 2 hours. Hence, to approach truly large scale experiments, the authors believe that implementations would likely come from the field of parallel algorithms.

Our analysis of the minimum haplotype tagging problem (MHT) relies on a relationship we establish to the minimum set cover problem (MSC). Exploiting this relationship, we can relate variants of the MSC problem to MHT. This we can do as we describe an algorithm that transforms a MHT instance into a MSC instance, solves this instance, and transforms this solution into a solution of the MHT problem. We can form variants of this algorithm by substituting particular variants of the MSC algorithm in this process. Below we list MSC variants, their known approximation properties, and if substituted into our algorithm schema, the corresponding resulting MHT problem that it solves.

• Minimum Exact Cover. This problem is approximable within 1 + ln *m *[12] and the associated tagging problem is to find a minimum set of SNPs such that the sets of haplotype pairs each of the SNPs distinguishes are as disjoint as possible.

• Maximum Coverage by at most *k *sets. This problem is approximable within *e*/(*e *- 1) [13] and not approximable within *e*/(*e *- 1) - *O*(1) [14] for *e *> 1. The associated tagging problem is the problem of discerning a maximum number of haplotypes with at most *k *SNPs.

• Minimum *k*-redundant Coverage is the problem of creating a minimum cover such that each element is covered at least *k *times. This problem is approximable within *b *- *k *+ 1 [15] for a constant *b*. The associated tagging problem is the problem of requiring each SNP in a minimum tagging set to discern between at least *k *pairs of haplotypes.

• Minimum Diameter Coverage is the problem of finding a cover where the greatest distance between two elements in the cover is minimal. This problem is in general not approximable within a constant, but approximable within 2 if the distance measure observes the triangle inequality and no better approximation is possible [16]. The associated tagging problem is to find a tagging set of SNPs such that the maximal pairwise distance is minimal.

• Minimum Cluster Diameter Coverage is the problem where we assume that the cover can be partitioned into *k *clusters and the objective is to minimize the greatest distance between two elements in the same cluster. This problem is approximable within 2 for any fixed *k *and upper bound of cluster size *l *if the distance measure satisfies the triangle inequality [16]. The associated tagging problem is to find *k *disjoint sets of maximally *l *SNPs such that the greatest distance between two SNPs in the same set is minimized.

• Maximum Dispersion Cover is the problem of finding a cover that maximizes the min imum pairwise distance between elements in the cover. This problem is NP-hard and assuming *P *≠ *NP*, no polynomial time approximation with bounded error guarantee exists [16]. The corresponding tagging problem is to find a set of SNPs that lets us discern between all haplotypes such that the minimum distance between two of these SNPs is maximized. We see that this problem is the most difficult to solve of all the problems considered in this exposition.

All these variations can be implemented by substituting the corresponding cover algorithm for the greedy set cover algorithm in the MHT algorithm schema. Approximation bounds and non-approximability results for the above variations can be used to establish the same for the resulting variation of the haplotype tagging problem analogously to what we do for the minimum set cover problem.

## Conclusion

Although the haplotype tagging problem is hard, it can be approached with a simple, fast and practical algorithm. The contribution of this work is not only yet another fast and simple algorithm for the tagging problem, but also the proof that this algorithm delivers a solution with the asymptotically best error bound possible.

Furthermore, the algorithm schema we present via the connection to the minimum set cover problem is applicable to not only the original problem as it is defined in our analysis, but is applicable to a family of related problems, which address problems presented in published research.

As the algorithms presented are asymptotically optimal with respect to approximation bounds, meaning that solution quality cannot be significantly improved upon in polynomial time, efforts in tackling truly large scale problem instances should concentrate on distributing the computational efforts in parallel.

## Methods

Let *H *= {*h*_1_, *h*_2_,..., *h*_*n*_} be a set of haplotypes with associated SNP markers at positions *S *= {1, 2,..., *m*}, and let *S' *be a subset of *S*. We define *h*_*i*_[*S'*] to be the string consisting of marker values of haplotype *h*_*i *_found at the positions in *S'*. We can view *H *as an *n *× *m *matrix over the set of possible SNP alleles values. Assuming that they are bi-allelic, *H *becomes a Boolean matrix. The problem of discerning a set of haplotypes by a minimum cardinality set of SNPs, alternatively the problem of predicting SNPs for a set of haplotypes using a minimal set of SNPs, which we will call the minimum haplotype tagging problem (MHT), can be formulated as follows.

**Problem 1 *(MHT) ****Let H, S and h_i _be as above. Find a minimum cardinality set S' *⊆ *S such that h*_*i*_[*S'*] = *h*_*j*_[*S'*] *implies i *= *j*.

We now define some formal concepts and present results that we will use in the analyses that follow.

### Formal concepts

We formally define a minimization problem  as a **3**-tuple (*p*, *s*, *m*) where

• *p *is the polynomial time computable characteristic function of the set of problem instances ,

• *s*(*x*) is the set of feasible solutions for instance *x *and its characteristic function is computable in polynomial time for any *y *such that |*y*| ≤ *O*(|*x*|^*q*^) for some *q *∊ **N**,

• *m *is the polynomial time computable natural number measure we wish to minimize.

In other words, the problem  is: given instance *x *such that *p*(*x*) = 1, find *y *∊ *s*(*x*) such that *m*(*y*) ≤ *m*(*z*) for any *z *∊ *s*(*x*). We then let m*(*x*) = *m*(*y*) denote the optimum value for instance *x*.

The problem of deciding whether a given tuple (*x*, *k*) is in *L*() = {(*x*, *k*)|*p*(*x*) = 1 ∧ m*(*x*) ≤ *k*} we will call the decision problem associated with , and, overloading notation slightly, denote by *L*().

Let *f *and *g *be two functions, and let  and  be two problems. If for all *x *∊  we have that *f*(*x*) ∊  and for all *y *∊ (*f*(*x*)) we have that *g*(*x*, *y*) ∊ (*x*) we say that the tuple (*f*, *g*) is a *reduction *from  to . If both *f *and *g *are computable in polynomial time, we write  ≤_*p *_ and call (*f*, *g*) a polynomial time reduction. Note that *g *is the identity on the solution for a reduction between decision problems.

In the following, we will rely on the result that an optimization problem  as defined above is NP-hard if the associated decision problem is NP-complete [[17], Theorem 1.2], and this can be established by finding a polynomial time reduction from a known NP-complete problem.

We now turn to the approximation properties of minimization problems. For instance *x *and solution *y *of minimization problem , we define the performance ratio of *y *and the optimal to be



and say that *y *∊ *s*(*x*) is an (*x*, *y*)-approximate solution for instance *x*. Let  be an algorithm that computes (*x*) ∊ *s*(*x*) for instance *x *of . We say that  is an approximation algorithm and  is an *r*(|*x*|)-approximation algorithm if (*x*, (*x*)) ≤ *r*(|*x*|) for all instances *x *such that *s*(*x*) ≠ *∅*. The class of optimization problems for which there exists a polynomial time *r*-approximation algorithm where *r *is a constant is called APX.

**Definition 1 ***[*[17], *Definition 8.3] Let **to **be two optimization problems as defined above, and let f and g be two functions. Let x be any instance x *∊ *and y any y *∊ (*f*(*x*)) *and enumerate requirements on f and g as follows*.

*1. f*(*x*) ∊ *and g*(*x*, *y*) ∊ (*x*) *are computable in polynomial time*,

*2. * (*x*) ≠ *∅ *⇒  (*f*(*x*)) ≠ *∅*

*3. * (*f*(*x*), *y*) ≤ *r *⇒  (*x*, *g*(*x*, *y*)) ≤ 1 + *α*(*r *- 1) *for constant α *≥ 1 *and constant rational r *> 1.

*If there exist functions f and g that fulfill requirements 1, 2, and 3 then the tuple *(*f*, *g*, *α*) *is an AP-reduction from **to **and we write * ≤_AP _.

Using AP-reductions, we can define completeness and hardness for classes of approximation problems analogously to how we do for problem complexity classes. In particular, a problem  is said to be APX-hard if  ≤_AP _ for all  in APX. If  itself is in APX, we say that  is APX-complete. By transitivity of AP-reductions, given  ≤_AP _, we have that  is APX-hard if  is APX-complete. We will rely on this result in the analyses that follow.

#### Analysis roadmap

We will proceed in the analysis with two goals in mind.

• Proving NP-hardness, and

• proving approximation bounds.

Our strategy is to "bracket" the problem of interest in a sequence of problems. As we choose the sequence endpoints such that we know the desired properties of these, we use reductions to propagate these results to the problems of interest.

We will prove NP-hardness of Problem 1 by reduction from the Minimum Set Cover problem (MSC). In fact, *L*(*P*_*MSC*_) ≤_*p *_*L*(*P*_*MHT*_) ≤_*p *_*L*(*P*_*MSC*_). In order to prove the approximation properties we show that the reductions used in the NP-hardness proofs can be used to construct AP-reductions. We show that *L*(*P*_*MSC*_) ≤_AP _*P*_*MHT *_≤_AP _*P*_*MSC *_with *α *= 1. We further show that the existence of a bounded version of MSC that is in APX naturally leads to the existence of APX version of MHT. Since the bounded MSC problem is known to be APX-complete, we have that the bounded version of MHT is APX-complete. Requirement 3 in Definition 1 lets us conclude that a lower approximation bound for MSC can be used via the AP reductions to produce a lower approximation bound for MHT as well.

### The optimization problems

The two problem formulations below are such that all instances can be seen as an *n *× *m *binary matrix *B *and the measure *m *for a feasible solution *I *is defined as *m*(*I*) = |*I*|. Hence, in the following, the problem definitions only consist of the specification of the function s for the instance *B*.

We can reformulate the "Minimum Haplotype Tagging" Problem 1 as follows.

**Problem 2 *(MHT) ****s*(*B*) = {*I *⊆ {1, 2,..., *m*}|*b*_*i *_≠ *b*_*j *_⇒ *b*_*i*_[*I*] ≠ *b*_*j*_[*I*]}

The following problem is the "Minimum Hitting Set" problem which we will use as a known starting point in our analyses.

**Problem 3 *(MSC) ****s*(*B*) = {*I *⊆ {1, 2,..., *n*}|∀_*j *_∊ {1, 2,..., *m*} ∑_*i *∊ *I*_*b*_*ij *_> 0}.

Johnson [12] presents a greedy 1 + ln *m*-approximation algorithm for MSC(*B*). Furthermore, if we bound *m *from above by a constant *K*, the MSC problem becomes APX-complete and approximable by [18].

### NP-hardness

**Lemma 1 ***L*(MSC) ≤_*p *_*L*(MHT) ≤_*p *_*L*(MSC).

*Proof*: (sketch) We will in the following represent the reduction from *L*() to *L*() by two polynomial time computable functions *f *and *g *such that



*L*(MSC) ≤_*p *_*L*(MHT): Let the Boolean *n *× *m *matrix *B *be the transposed of an instance of the MSC problem such that *s*(*B*^*t*^) ≠ *∅*. Note that we can represent the integers between 0 and *n *- 1 using a bit-string of minimal length [log_2 _*n*]. Let *b*(*i*, *j*) denote the *jth *bit of this representation of the integer *i*, 0 ≤ *i *<*n*. Let *B' *=  be the (2*n*) × (*m *+ [log_2 _*n*]) matrix constructed as follows.



Then *f*_*MHT*_(*B*) = *B' *is an 2*n *× *m *+ [log_2 _*n*] instance of the MHT problem. We now have to show the existence of *g*_*MHT*_. Define . Note that any solution *S' *of the MHT problem has to contain an element of *d*(*i*, *j*) for any pair *i *≠ *j*. From the construction of *B' *we can see that any solution *S' *has to contain *S" *= {*i*|*m *<*i *<*m *+ [log_2 _*n*]} and that *S" *is sufficient for any (*i*, *j*) such that *i *mod *n *≠ *j *mod *n*. Disregarding the cases of *i *= *j*, we are left with the cases 1 ≤ *i *≤ *n *and *j *= 2*i*. For these cases we have that *d*(*i*, 2*i*) = {*k*|*b*_*ik *_= 1}. We then have that *S' *has to contain *S" *for (*i*, *j*) such that *i *mod *n *≠ *j *mod *n*, and *S' *has to intersect *d*(*i*, *j*) = {*k*|*b*_*ik *_= 1} for 1 ≤ *i *≤ *n *and *j *= 2*i*, the latter meaning *S' *has to contain a solution of the MSC problem instance *B*^*t*^. Noting that the *d*(*i*, 2*i*) ∩ *S" *= *∅*, we see that as |*S'*| = |*S"*| + *k *we have that *g*_*MHT*_(*x*, *k*) = *k *- [log_2 _*n*] is the *g*_*MHT *_we are looking for. As both *f*_*MHT *_and *g*_*MHT *_are computable in polynomial time, we have the sought reduction.

*L*(MHT) ≤_*p *_*L*(MSC): Let the *n *× *m *matrix *B *be an instance of the MHT problem. Let *d*(*i*, *j*) = {*k*|*b*_*ik *_≠ *b*_*jk*_}. Let *C *be the *n' *× *m *Boolean matrix representation of  = {*d*(*i*, *j*) ≠ *∅*|1 ≤ *i *<*j *≤ *n*}, where *n' *< (*n*^2 ^- *n*)/2. Further, let *f*_*MSC*_(*B*) = *C*^*t*^, and let *g*_*MSC*_(*x*, *k*) = *k*. It is again not hard to convince oneself that *s*_*MHT*_(*B*) = *s*_*MSC*_(*f*_*MSC*_(*B*)). Then (*f*_*MSC*_, *g*_*MSC*_) is the sought reduction.

As we know that *L*(MSC) is NP-complete we have by Lemma 1 that L(MHT) is NP-hard. We also have that any solution of MHT is of size at most the size of the instance it is a solution of. This lets us conclude that there exists a polynomial time verifier for *L*(MHT) languages, meaning that *L*(MHT) is in NP and hence NP-complete by the above lemma. This in turn lets us state the following theorem.

**Theorem 2 ***L*(MHT) *is NP-complete and MHT is NP-hard*.

### Approximability properties

**Lemma 2 ***Let **and **be two minimization problems and let f and g be two functions that satisfy requirements 1. and 2. from Definition 1. Let h*(*x*) ≥ 0 *for x *∊ . *If *(*g*(*x*, *y*)) = (*y*) - *h*(*x*) *for all x *∊ *and for all y *∊ (*f*(*x*)), *then *(*f*, *g*, 1) *is an AP-reduction from **to *.

*Proof*: We only need to check the requirement



We have that both  and  are minimization problems. Then,



Since (*x*) ≤ (*g*(*x*, *y*) we have that  which in turn means that



This in turn means that



We can conclude



A similar argument can be used to prove the general case. i.e., not only for minimization problems.

**Lemma 3 **MSC ≤_AP _MHT ≤_AP _MSC

*Proof*: Recall *f*_*MHT *_and f_*MSC *_constructed in the proof of Lemma 1. We see from their construction that requirements 1. and 2. in Definition 1 all are met by the pairs (*f*_*MHT*_, *g*_*MHT*_) and (*f*_*MSC*_, *g*_*MSC*_) if we let *g*_*MHT*_(*x*, *y*) = *y *- *S"*, where S" is defined as in the discussion of the reduction from *L*(*MSC*) to *L*(*MHT*) in the proof of Lemma 1, and *g*_*MSC*_(*x*, *y*) = *y*.

Let *h*_*MHT*_(*x*) = [log_2 _*n*] and let *h*_*MSC*_(*x*) = 0. We then see that both reductions together with the corresponding *h *match the conditions of Lemma 2, which completes our proof.     

#### The good news

**Theorem 3 ***MHT is approximable within *1 + ln().

*Proof*: The theorem follows from that if *B *is an *n *× *m *instance of MHT, then *f*_*MSC*_(*B*) is the *m *× *n'*, *n' *≤ (*n*^2 ^- *n*)/2 instance of MSC. MSC is known to be approximable within 1 + ln *m *for *n *× *m *instances, and since we know from Lemma 3 that we have an AP-reduction from MHT to MSC with *α *= 1 we have that MHT is approximable within 1 + ln().     

#### The bad news

Let MHT' be the version of MHT where we bound *n *from above by a constant.

**Theorem 4 ***MHT' is APX-complete*.

*Proof*: From Lemma 3 it follows that MSC' ≤_AP _MHT' ≤_AP _MSC', where MSC' is the version of MSC where we bound *m *by a constant for *n *× *m *instances. From Theorem 3 it follows that MHT' is in APX, and as MSC' is known to be APX-complete [18], the theorem follows.     

**Theorem 5 ***It is NP-hard to approximate MHT within c*ln(*n*/2) *for any constant c *> 0 *and MHT is not approximable within *(1 - *ε*)ln(*n*/2) *for any ε *> 0 *unless *NP ⊂ DTIME(*n*^log log *n*^).

*Proof*: We know [19] that it is NP-hard to approximate MSC within *c*ln *m *for any constant *c *> 0. We also know [14] that MSC is not approximable within (1 - *ε*)ln(*n*) for any constant *ε *> 0 unless NP ⊂ DTIME(*n*^log log *n*^). From the proof of Lemma 3, we know that MSC ≤_AP _MHT with *α *= 1, meaning that



If *x *is an *m *× *n' *instance of MSC, then *f*_*MHT*_(*x*) as defined in the proof of Lemma 3 is a 2*n' *× (*m *+ [log_2 _*n*]) instance of MHT. If we have a *r*(*n*)-approximation algorithm for MHT, we have a *r*(2*n'*)-approximation algorithm for MSC, and the theorem follows.     

### Algorithms

We present here two polynomial time algorithms for solving the MHT problem that exhibit the approximation bound found above. The first algorithm is based on the transformation to the minimum set cover problem. This allows us to guarantee the performance bounds. The second algorithm does not compute a transformation explicitly, but accomplishes the same effects directly. The second algorithm is much simpler to implement, uses significantly less space and has the same asymptotic running time as an optimal implementation of the first.

The advantage of the algorithm with the explicit transformation to the minimum set cover problem is that it lets us produce algorithms for a family of haplotype tagging problems as presented in Section.

Let *B *be an *n *× *m *instance of the MHT problem such that each row is unique. The first algorithm is essentially applying an implementation of *f*_*MSC *_followed by the application of an algorithm for the set cover problem. To understand how this works, consider that each set in the collection represented in matrix form by *f*_*MSC*_(*B*) is associated with one column (representing a SNP) in *B*. We can form (*n*^2 ^- *n*)/2 pairs of row indices such that their respective rows in *B *are different. Let each of these pairs be associated with a unique identifier. Each set in *f*_*MSC*_(*B*) contains the unique identifiers of the row pairs that differ in the associated column in *B*. The objective of the MHT problem is to find a minimum number of columns (equivalently sets in *f*_*MSC*_(*B*)) such that they together discern between all pairs of rows (cover all the unique row pair identifiers).

Johnson's 1 + ln *m *greedy approximation algorithm [12] iteratively selects the set that covers the most uncovered elements and adds it to the initially empty solution. This is done until all elements are covered. It is well known that a greedy algorithm for the unweighted set cover problem on the collection  can be implemented to run in *O*( ≤ *O*(*m*(*n*^2 ^- *n*)/2) time [[20], Exercise 37.3.3]. We state without proof that it is also not hard to implement *f*_*msc *_to run within the same bounds. Hence the promised first algorithm consisting of running the implementation of *f*_*MSC *_and applying the greedy set cover algorithm has running time *O*( ≤ *O*(*m*(*n*^2 ^- *n*)/2) and exhibits the promised approximation bound. We will in the following refer to this algorithm as MHT.

The second algorithm is based on the following observation. Selecting the set that covers the most uncovered elements in the algorithm above is equivalent to selecting the column that discerns between the most pairs of previously undiscerned rows. Hence we can achieve the same effect as the first algorithm by recursively partitioning the set of rows in *B *by at each iteration selecting the column that refines the partition the most. This more direct algorithm we will call D-MHT. Pseudo code for it is given below.

The function delta(*i*, *L*) computes the number of pairs of rows in *B *indexed by elements in *L *that can be discerned by using column *i *in *B*, i.e., delta(*i*, *L*) = *s*(*l *- *s*) where *s *= ∑_*j *∊ *L*_*b*_*ji *_and *l *= |*L*|. The function part(*b*, *L*) splits *L *into two lists, one that contains the indices of rows in *B *that have a 1 in column *b *and one that contains the indices of rows that have a 0 in column *b*. The function append(*LL"*, *LL'*) appends *LL" *to the list *LL'*. Note that delta(*i*, *L*) and part(*b*, *L*) run in *O*(|*L*|) time, and that append() runs in *O*(1) time. We note that these running times can also be achieved for the general case of *B *being a matrix D-MHT(*B*)

(1)   *P *← *∅*

(2)   *LL *← NULL

(3)   *U *← {1, 2,..., *m*}

(4)   insert((1, 2,..., *n*), *LL*)

(5)   **while **|*LL*| <*n*

(6)      *a *← 0

(7)      *b *← 0

(8)      **foreach ***i *∊ *U*

(9)         *s *← 0

(10)         **foreach ***L *∊ *LL*

(11)            *s *← *s *+ delta(*i*, *L*)

(12)         **if ***s *> *a*

(13)            *a *← *s*

(14)            *b *← *i*

(15)      *P *← *P *⋃ {*b*}

(16)      *U *← *U *- {*b*}

(17)      *LL' *← NULL

(18)      **foreach ***L *∊ *LL*

(19)         *LL" *← part(*b*, *L*)

(20)         append(*LL"*, *LL'*)

(21)      *LL *← *LL'*

(22)   **return **(*P*)

containing natural numbers, with the size of the range of values in each row being within *n*, making the D-MHT algorithm a general partitioning algorithm useful for among others pattern discovery.

The loop 5–21 in D-MHT is performed *p *= |*P*| times. As one element gets added to *P *for each iteration of this loop, the loop 8–14 gets executed  = *p*(2*m *- *p *+ 1)/2 times. As delta(*i*, *L*) is *O*(|*L*|), and *LL *is a partition of *n *numbers, we have that loop 10–11 is ∑_*L *∊ *LL*_*O*(|*L*|) = *O*(*n*). Hence line11 is *O*(*np*(2*m *- *p *+ 1)/2). Similarly, 20 is *O*(*np*(2*m *- *p *+ 1)/2) and 21 is *O*(*n*). It follows that the running time of the algorithm is



where *p *= |*P*|. We see that *p *≤ min(*n*, *m*) by the fact that each column chosen splits at least one element in *L *and that for *n *elements, the maximal number of times just one element in *L *can be split is *n*. Also, we cannot choose more than *m *columns. Also, *O*((2*m *- *p *+ 1)) is monotonously increasing with *p *≤ *m*. If *m *≤ *n *we have that



If on the other hand *m *> *n*, then we have that



Now assume that *m *> *n *> 1, and look at the ratio of the running times of D-MHT and MHT respectively. This ratio is given as



Again using that *m *> *n*, we see that



for *n *> 1. Indeed, as *n *→ ∞ the above approaches 2.

Now assume that 1 <*m *≤ *n*. Then we have that the same ratio is



The ratio above is maximal when *m *= *n*, 1 when *m *= *n *- 2, and we see a linear increase of the reciprocal of the ratio with the quantity *n *- *m*. Knowing that *p *≤ min(*n*, *m*) yields



We hence conclude that the direct algorithm has the same worst case asymptotic running time as the one based on the optimal implementation of the minimum set cover problem. The direct algorithm is simpler to implement, might run faster in short solution cases, and has significantly smaller space requirements as we do not need to construct the set cover instance. Also, it might be of interest to weight SNPs. While including weights is trivial in the D-MHT algorithm, implementing an *O*(*mn*) version of the minimum weighted set cover problem can prove to be a difficult task. This is because the unweighted algorithm depends on a data structure that allows remove-max, remove, and insert in constant time but depends on keys to lie in a known, finite, and small set. However, a *O*((*mn*) ln(*mn*)) implementation is readily available.

A third algorithm can be constructed by applying the best suited of the two algorithms presented above. The analysis suggests that one could choose MHT over D-MHT whenever *m *> *n *- 2.

## Authors' contributions

SAV analyzed the approximation properties of the MHT problem and suggested the D-MHT algorithm. SD participated in the analysis of the running time complexities and the placement of the paper in context. LOM related the work to the context it is presented in. All authors wrote parts of the manuscript.
